# Techniques for transferring host-pathogen protein interactions knowledge to new tasks

**DOI:** 10.3389/fmicb.2015.00036

**Published:** 2015-02-02

**Authors:** Meghana Kshirsagar, Sylvia Schleker, Jaime Carbonell, Judith Klein-Seetharaman

**Affiliations:** ^1^School of Computer Science, Language Technologies Institute, Carnegie Mellon UniversityPittsburgh, PA, USA; ^2^Metabolic and Vascular Health, Warwick Medical School, University of WarwickCoventry, UK; ^3^Molecular Phytomedicine, Institute of Crop Science and Resource Conservation, University of BonnBonn, Germany

**Keywords:** protein interaction prediction, host pathogen protein interactions, plant pathogen protein interactions, machine learning methods, transfer learning, kernel mean matching

## Abstract

We consider the problem of building a model to predict protein-protein interactions (PPIs) between the bacterial species *Salmonella* Typhimurium and the plant host *Arabidopsis* thaliana which is a host-pathogen pair for which no known PPIs are available. To achieve this, we present approaches, which use homology and statistical learning methods called “transfer learning.” In the transfer learning setting, the task of predicting PPIs between *Arabidopsis* and its pathogen *S*. Typhimurium is called the “target task.” The presented approaches utilize labeled data i.e., known PPIs of other host-pathogen pairs (we call these PPIs the “source tasks”). The homology based approaches use heuristics based on biological intuition to predict PPIs. The transfer learning methods use the similarity of the PPIs from the source tasks to the target task to build a model. For a quantitative evaluation we consider *Salmonella*-mouse PPI prediction and some other host-pathogen tasks where known PPIs exist. We use metrics such as precision and recall and our results show that our methods perform well on the target task in various transfer settings. We present a brief qualitative analysis of the *Arabidopsis*-*Salmonella* predicted interactions. We filter the predictions from all approaches using Gene Ontology term enrichment and only those interactions involving *Salmonella* effectors. Thereby we observe that *Arabidopsis* proteins involved e.g., in transcriptional regulation, hormone mediated signaling and defense response may be affected by *Salmonella*.

## 1. Introduction

Understanding the workings of plant responses to pathogens is an important fundamental questions that also has enormous economic importance due to the role of pathogens in food production and processing. While “classical” plant pathogens cause crop losses during production by impacting on plant health, processing of plant-based food can lead to contamination by opportunistic pathogens. It is becoming increasingly supported by experimental evidence that some human bacterial pathogens can colonize plants and cause disease (Kirzinger et al., 2011). *Salmonella* is one of these bacterial species with extremely broad host range that infects not only animals, but also plants (Hernandez-Reyes and Schikora, 2013). Evidence increases that *Salmonella* can utilize plants as alternative host and can be considered as a bona fide plant pathogen. In this respect it has been reported that (a) *Salmonella* actively invades plant cells, proliferates there and can cause disease symptoms (Schikora et al., 2008; Berger et al., 2011) (b) the plant recognizes *Salmonella* and plant defense responses are activated (Iniguez et al., 2005; Schikora et al., 2008) and (c) that functional Type Three Secretion Systems (TTSS) 1 and 2 are important for *Salmonella* pathogenicity in plants with respect to bacterial proliferation and suppression of plant defense responses (Iniguez et al., 2005; Schikora et al., 2011; Shirron and Yaron, 2011). *Salmonella* TTSS-1 and 2 encode proteins, so called effectors, which are known to be translocated into the animal host cell in order to manipulate host cell mechanisms mainly via PPIs (Schleker et al., 2012). Hence, it may be assumed that *Salmonella* utilizes the same proteins during its communication with animals and plant. However, the details of this communication are not known. A critical component of the communication between any host and its pathogen are PPIs. However, the infection of plants by *Salmonella* is only a nascent field, so there are no known PPIs for *Salmonella* with any plant reported yet. Even for the well established pathogen-host pair, *Salmonella*-human, relatively few interactions are known (Schleker et al., 2012). Only 62 interactions between *Salmonella* and mostly human proteins (some *Salmonella* interactions involve other mammalian species, such as mouse and rat) are known to date. Because there exists no plant-*Salmonella* interactions data, we need to rely on computational methods to predict them [reviewed in the accompanying paper (Schleker et al., 2015)].

**Figure 1:**
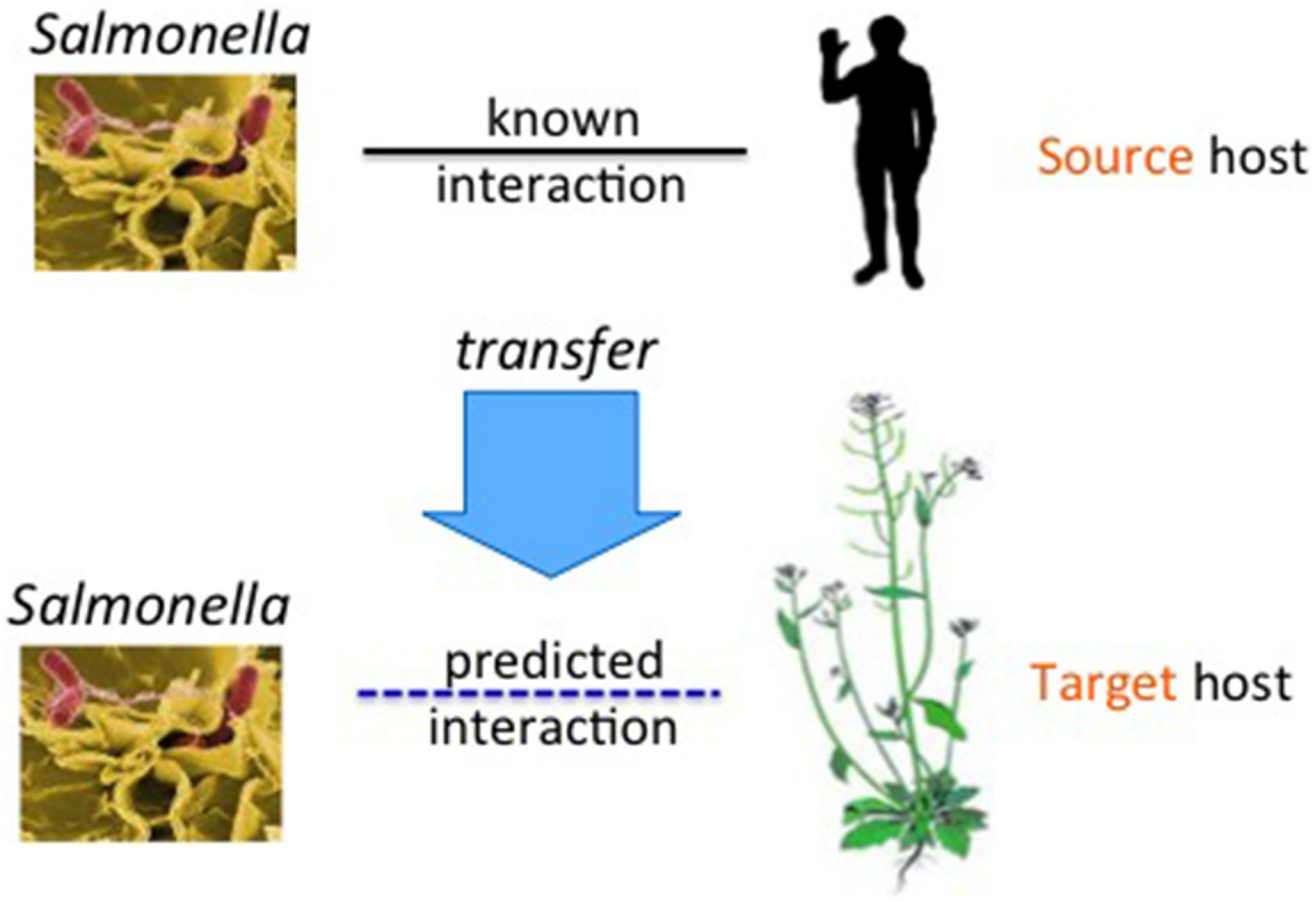
**Transfer of PPIs from the source host (for ex: human) to another host, the target host (for example *Arabidopsis*), for the common pathogen, *Salmonella***.

In this paper, we describe techniques to build computational models to predict interactions between the model plant, *A. thaliana*, and *S*. Typhimurium. Since there is no labeled data of this host-pathogen pair available, we aim to transfer knowledge from known host-pathogen PPI data of other organisms. We use various statistical methods to build models for predicting host-pathogen PPIs. In each case, we cast the PPI prediction problem as a binary classification task, where given two proteins the goal is to learn a function that predicts whether the pair would interact or not. We derive features on every protein pair using protein sequence data. Each host-pathogen PPI prediction problem is considered as one task. Figure 1 shows our problem setting. The upper host-pathogen task with *Salmonella* as pathogen and human as the host is the *source* task. The lower task is the target task. The arrow shows the direction of knowledge transfer.

In order to transfer knowledge from one organism to another, we need to utilize some measure of similarity between them. This similarity can be defined between smaller units such as individual proteins or genes from the organisms or higher level units. The higher the similarity, the greater the information transfer between them. Hence the notion of similarity is very critical to the results we obtain from such a transfer based method and should be biologically motivated. Our methods enable the transfer of knowledge using the following mechanisms:

- We use the structural similarity between the individual proteins of the two hosts measured using protein sequence alignment. This follows from the biological intuition that structurally similar proteins in two different organisms are very likely to have similar functions. Hence a pathogen that wants to disrupt a specific function will target structurally similar proteins in different hosts.
- Interactome-level similarity, comparing the human PPI graph with the plant PPI graph. Any biological process in an organism involves the participation of several proteins and more importantly the interactions between these. By comparing the interactomes of different hosts, we are comparing them at the biological process-level. The components of the two graphs that are highly similar will most likely correspond to similar processes in the two organisms.
- Distributional similarity between the protein pairs: here, we identify which of the human-*Salmonella* protein pairs are the most similar (hence most relevant) to the plant-*Salmonella* protein pairs. This similarity is computed using the features of the protein-pairs. Since it is distributional similarity, it involves a comparison over all protein pairs from both organisms. Only the most relevant human-*Salmonella* protein pairs are used to build a model.

The main contributions of this paper are:

1. We present methods that combine known PPIs from various sources to build a model for a new task
2. We evaluate our methods quantitatively and our results show the benefits in performance that are possible if we incorporate the similarity information discussed in the previous paragraphs
3. We present the first machine learning based predictions for plant-*Salmonella* PPIs.

In the rest of the paper, we start by describing the host-pathogen PPI datasets we use in Section 2, followed by a detailed description of our methods in Section 3 and a quantitative and qualitative analysis of the results in Section 5.

## 2. Source tasks

As source tasks we used the known PPIs between various other hosts and pathogens. Many of these interactions were obtained from the PHISTO (Tekir et al., 2012) database which reports literature-curated known interactions. For PPIs between human and *Salmonella* we use the manually literature-curated interactions reported in Schleker et al. (2012). Please note that all of these interactions come from biochemical and biophysical experiments. The details of the dataset used in each approach are shown in Table 1 and they are available for download from http://www.cs.cmu.edu/~mkshirsa/data/frontiers2014/data.zip. Our first approach is a rule-based approach and it uses human-*Salmonella* PPIs from two sources: the 62 experimentally generated PPIs reported in Schleker et al. (2012) and the predicted PPIs from Kshirsagar et al. (2012). Please note that this is the only method that uses any predicted PPIs as “ground truth.” All other methods discussed in subsequent sections of this paper do not use any predicted PPIs as source. They use only PPIs validated experimentally by biochemical and biophysical methods.

**Table 1:** **Datasets used in the various approaches, their sizes and the appropriate citations**.

**Approach(es)**	**Source task(s)**	**Number of interactions**	**Citation for interactions data**	**Feature set**
1. Homology based	Human-*Salmonella* known PPI Human-*Salmonella* predictions	62 190,868	Schleker et al., 2012^*^ Kshirsagar et al., 2012	No feature set. Heuristics are used to infer interactions
2. T-SVM^#^	Human-*Salmonella* known PPI	62	Schleker et al., 2012^*^	(a) Protein sequence k-mers
				(b) Gene expression (from GEO)
				(c) GO term similarity
3. KMM^†^-SVM	Human-*Salmonella* known PPI	62	Schleker et al., 2012^*^	
	Human-*Francisella tularensis*	1380		
	Human-*E.coli*	32		
	*A. thaliana* - *Agrobact. tumefaciens*	22	PHISTO^*^	Protein sequence k-mers
	*A. thaliana* - *E. coli*	15	(Tekir et al., 2012)	
	*A. thaliana* - *Pseudomonas syringae*	13		
	*A. thaliana* - *Synechocyctis*	23		

† KMM, Kernel Mean Matching;

# *SVM, Support Vector Machine; GO, Gene Ontology*.

* *This source reports PPIs validated experimentally by biochemical and biophysical methods*.

### 2.1. *Salmonella* species/strains considered

The source data that we use for human-*Salmonella* from Schleker et al. (2012) comes from two different strains: *Salmonella* Typhimurium strain LT2 and *Salmonella* Typhimurium strain SL 1344. One of our three approaches (row-1 of Table 1) uses human-*Salmonella* predicted PPIs. These predicted PPIs from Kshirsagar et al. (2012) contain *Salmonella* proteins from two additional strains: *Salmonella* enteritidis PT4 and *Salmonella* Typhi. From henceforth, for the sake of brevity, we will refer to proteins from all strains as *Salmonella* proteins. For *Salmonella* proteins, we used the UniprotKB database (The UniProt Consortium, 2014) to obtain all proteins from the various strains. For *Arabidopsis* thaliana proteins, we used the TAIR database (Lamesch et al., 2012).

## 3. Methods

In the previous section, we described the dataset used in our various approaches. We now describe the details of the methods we use.

### 3.1. Approach-1 : homology based transfer

In this approach, we use the sequence similarity between the plant and human protein sequences to infer new interactions. We use two techniques to predict interactions between plant and *Salmonella* proteins. The first technique uses plant-human orthologs and the second is based on plant-human homology (sequence alignment scores). Both techniques use two sources of interactions: true PPIs from Schleker et al. (2012) and predicted PPIs from Kshirsagar et al. (2012). Please note that this is the only method that uses any predicted PPIs as “ground truth.” All other methods discussed in subsequent sections of this paper do not use any predicted PPIs as source.

*Homologs and Orthologs*: Homologous pairs of genes are related by descent from a common ancestral DNA sequence. These can be either orthologs: genes that evolved from a common ancestral gene by speciation or paralogs: genes separated by the event of genetic duplication. We obtained orthologs from the InParanoid database (Ostlund et al., 2010). To find homologous pairs of proteins, we used BLAST sequence alignment with an *e*-value threshold of 0.01.

**Figure 2:**
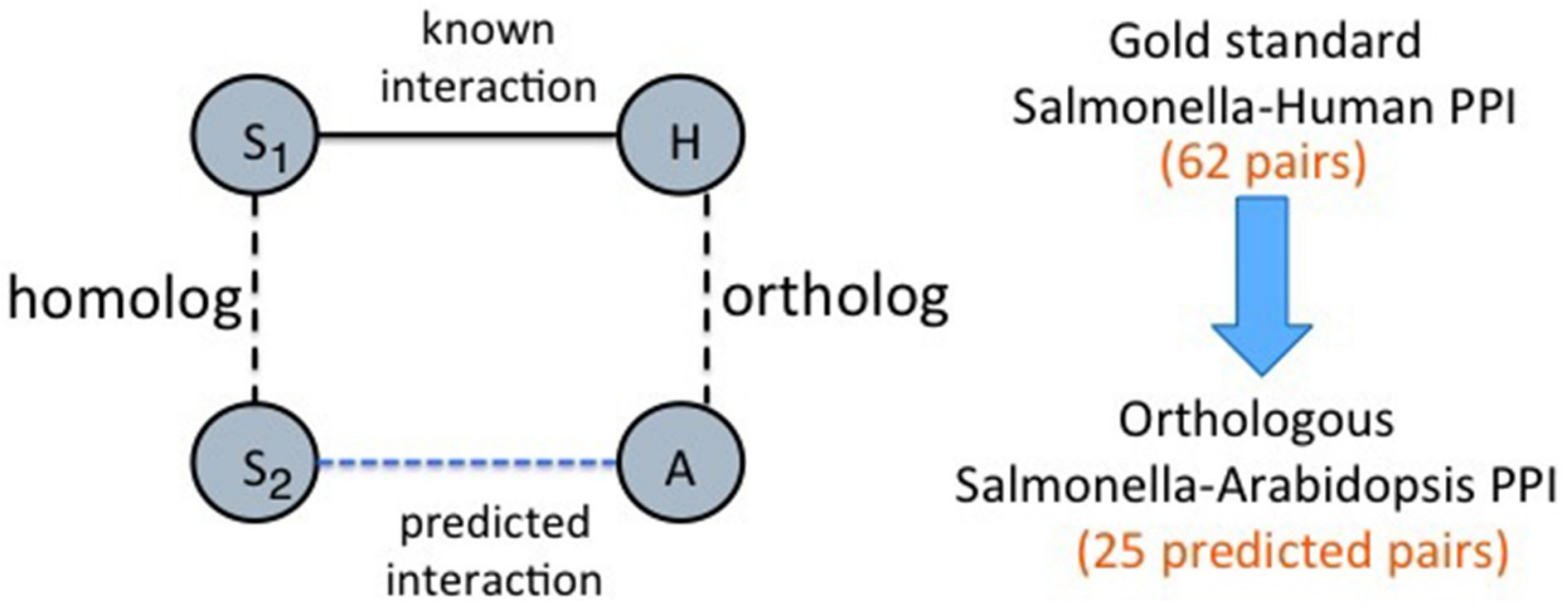
**Approach-1 (a) Ortholog based protein interaction inference**. “*S*_1_” represents a *Salmonella* protein and *S*_2_ is the homolog of *S*_1_ or *S*_1_ itself. *H* represents a human protein and *A* represents an *Arabidopsis* protein that is an ortholog of the human protein.

**Figure 3:**
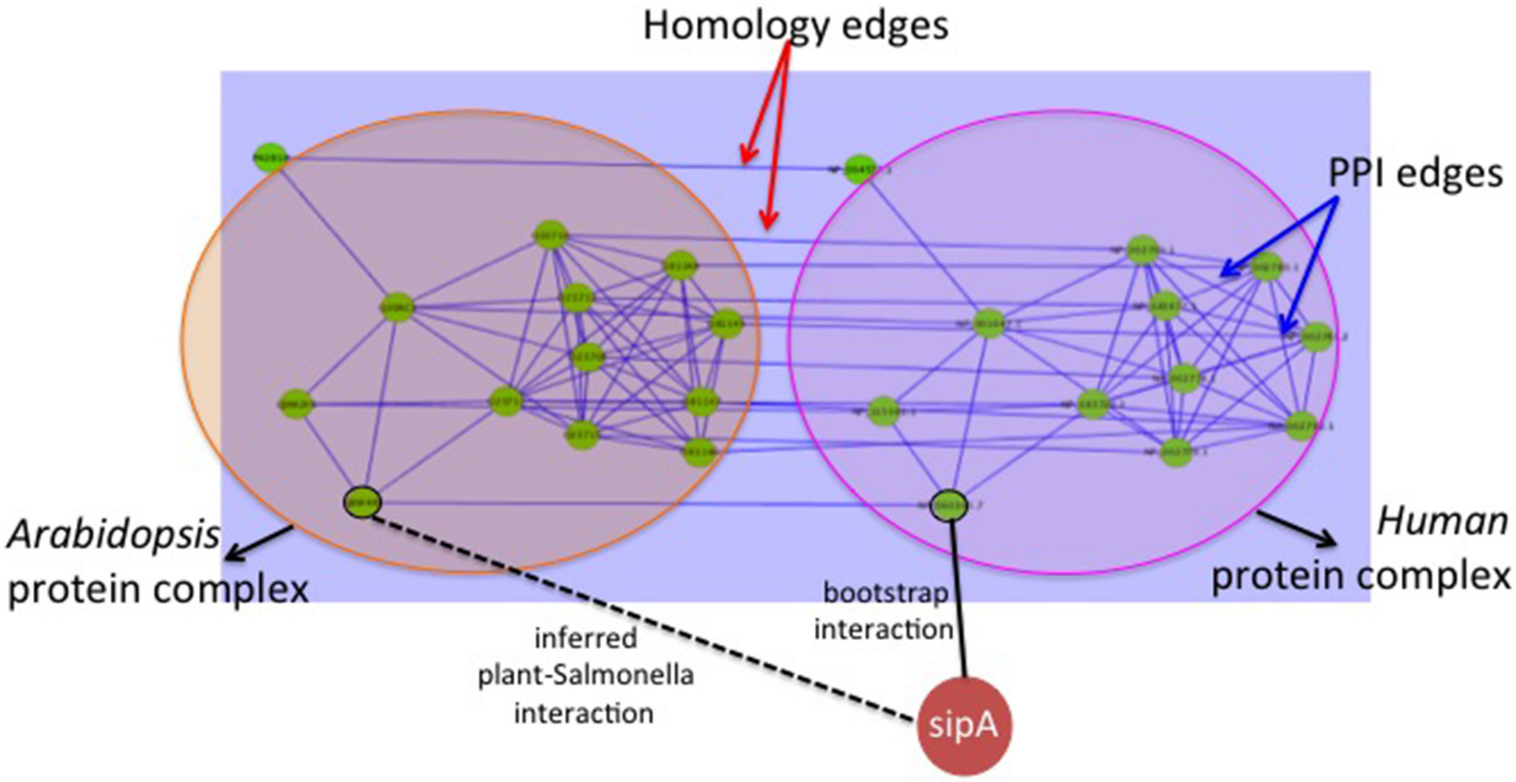
**Approach-1(b) Graph based interaction transfer**. The big circles show the two protein complexes found to be enriched by Network Blast : the *Arabidopsis* protein complex on the left, and the human protein complex on the right. The edges within a protein complex are the PPIs within the host organism. The edges connecting the two protein complexes (i.e., the two circles) are the homology edges. The solid line connecting *sipA* with a human protein node is a bootstrap interaction. We use this to infer the new plant-*Salmonella* interaction indicated by the dotted line.

- **Host ortholog based predictions**: We start with the known human-*Salmonella* PPIs. For each interaction, we search for an ortholog of the human protein in *Arabidopsis*. If one exists, we infer an interaction between the *Salmonella* and the *Arabidopsis* protein. This is similar to finding interologs, with the exception that we restrict ourselves to orthologs of the host protein rather than considering all possible homologs of both the host and pathogen proteins. Figure 2 illustrates this simple heuristic. There are 62 human-*Salmonella* PPIs in our dataset. Using this ortholog based inference for the host proteins, we obtained a total of 25 plant-*Salmonella* PPIs as some of the human proteins did not have any plant orthologs. The orthologous *Arabidopsis* proteins for the human proteins were obtained from the InParanoid database (Ostlund et al., 2010).
- **Host graph alignment based predictions**: This method uses homologs between the human and plant proteins. Since the set of known PPIs is very small (62 interactions), here we use them to generate “bootstrap” interactions. The known 62 PPIs are used to build a classifier using the method published in Kshirsagar et al. (2012) to generate a total of 190,868 human-*Salmonella* PPI predictions. These predicted PPIs form the “bootstrap” PPIs and will be used in a graph-based transfer approach. In this graph-based transfer method, we first align the PPI graphs of the two host organisms using NetworkBlast (Sharan et al., 2005). The human PPI network was obtained from the HPRD database (Prasad et al., 2009) and the plant-plant PPIs from TAIR database (Lamesch et al., 2012). The algorithm aligns the human PPI graph with the plant PPI graph using the pairs of homologous proteins between the two organisms. To find the homologous proteins, we used BLAST sequence alignment with an *e*-value threshold of 0.01. Next, we use NetworkBlast to find the graph components that are the most similar across the two graphs. We call them the “enriched components.” By comparing the interactomes of the two hosts, we are comparing them at the biological process-level. The components of the two graphs that are highly similar will most likely correspond to similar processes in the two organisms. NetworkBlast finds a total of 2329 enriched protein complex pairs between the two host organisms. Figure 3 shows one such enriched protein complex pair: the complex on the left is from *Arabidopsis* and the one on the right is from human. Using these we determine the plant proteins that are the most likely targets for the different *Salmonella* proteins as shown in the Figure 3.
  For each PPI between a human protein from an enriched protein complex, we infer an equivalent PPI between the corresponding plant protein and the *Salmonella* protein (example, *sipA* in the Figure 3). This filtering procedure gives us a final of 23,664 plant-*Salmonella* PPIs. The biological relevance for using the enriched graph components lies in the premise that clusters of similarly interacting proteins across the two organisms will represent biological processes that have been conserved in the two organisms. Hence, the proteins in these components are also likely to be conserved as pathogen targets.

### 3.2. Approach-2: transductive learning

This method considers the target task i.e., plant proteins while building a model. It provides a way of incorporating the target task information during model construction. Conventional inductive learning approaches such as the Support Vector Machine classifier use only the training examples to build a model. Transductive learning approaches also use the distribution of the unlabeled test examples. They jointly learn the labels on the test examples while minimizing the error on the labeled training examples. This often results in a good performance, as the classifier has additional information about the unseen test data. In our work here, we use transductive learning for transfer learning in particular the Transductive Support Vector Machine algorithm (T-SVM) (Joachims, 1999). The training examples are the source task examples, i.e., human-*Salmonella* protein interactions. We use the target task examples as the test data.

**Training negatives**: Since there are 62 known PPIs in the source task, we sample a set of random 6200 human-*Salmonella* protein pairs to maintain the positive:negative class ratio at 1:100.

**Figure 4:**
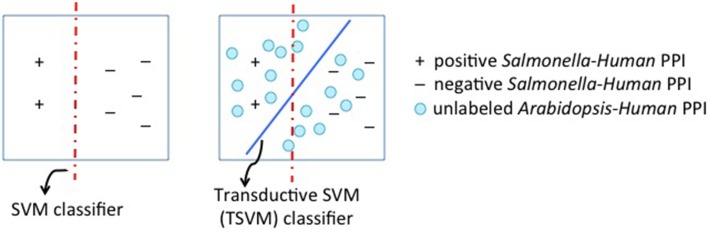
**Transductive Support Vector Machine (SVM) for transfer learning**. The first panel shows the conventional SVM classifier. The second panel shows T-SVM with circles representing unlabeled examples. We use examples from the target task i.e., *Arabidopsis*-*Salmonella* protein pairs as the unlabeled examples to influence the classifier boundary.

Figure 4 depicts this setting. This method thus builds a model by using data from both hosts. The optimization function of T-SVM jointly minimizes the training error on the known human-pathogen interactions and the label assignments on the unknown plant-pathogen interactions. The set of target examples can not be used entirely as it is very large and makes the T-SVM algorithm very computationally expensive. Hence we randomly sample 1 percent of the target dataset. For the T-SVM based algorithm to be effective, the kernel function that is used to compute the similarity between examples matters a lot. We use a homology-based kernel function that incorporates the BLAST similarity score between the proteins. Let *x^i^_s_* be the feature-vector representing a source task example: the protein pair < *s_s_*, *h_s_* > where *s_s_* is the *Salmonella* protein (i.e., the pathogen protein) and *h_s_* is the host protein. Let the target task example be the protein pair < *s_t_*, *a_t_* > where *a_t_* is the *Arabidopsis* protein; and the corresponding feature vector be *x^k^_t_*. The kernel function that computes the similarity between the given two pairs of proteins (i.e., their feature vectors) is defined as shown below.

$$\begin{array}{l} k(x_{s}^{i},x_{t}^{k})=\text{sim}(p_{s},p_{t})+\text{sim}(h_{s},a_{t})\text{ where}\\ \text{ sim}(m,n)=\text{normalized}-\text{BLAST}-\text{score}(m,n)\\ k(x_{s}^{i},x_{s}^{j})=dot(x_{s}^{i},x_{s}^{j})\text{ and }k(x_{t}^{i},x_{t}^{j})=dot(x_{t}^{i},x_{t}^{j}) \end{array}$$

The first equation is used in the case where the two protein pairs come from different tasks. We use homology-distance between the pathogen proteins and the host proteins to compute the kernel. The homology distance itself is simply the BLAST protein sequence alignment score. The next two equations show the computation when the examples both come from the same task. Here we simply take the dot product of the two feature vectors. This kernel is symmetric. The similarity between two sequences *sim*(*m*, *n*) is computed using the bit-score from BLAST sequence alignment, normalized using the sequence length of the larger protein. We used the SVM*^light^* package (Joachims, 2008) and incorporated our kernel function into it. The parameter tuning for T-SVM (the regularization parameter *C*) was done using cross validation on the PPIs where we have the true labels. We found *C* = 0.1 was the best setting. This best model is subsequently used to generate predictions on all *Arabidopsis*-*Salmonella* protein-pairs. The model outputs a score indicating the distance from the classifier hyperplane. A positive score indicates that the protein-pair is on the positive side of the hyperplane and hence closer to the known interacting protein-pairs. All such protein-pairs will be considered as potential interactions predicted by this model.

### 3.3. Approach-3: kernel mean matching

Our transfer learning scenario here consist of the following setting: multiple “source” tasks with small amounts of labeled data, a single “target” task with no labeled data. The first challenge is to pick the best instances from the source tasks, such that the resultant model when applied on the target task generates high confidence predictions. Toward this, we use the instance reweighting technique Kernel Mean Matching (KMM). The reweighted source task instances are used to build a kernelized support vector machine (SVM) model, which is applied on the target task data to get the predicted PPIs. This brings forth the second challenge—selecting appropriate hyperparameters while building a model for a task with no labeled data. For simplicity we also use the same set of features across all tasks (protein sequence features). However the data distribution will be different across tasks due to the different organisms involved.

This approach is based on instance-transfer where the goal is to pick from each of the source tasks, the most relevant instances w.r.t the target task. We use a two-step process: (1) the first step does the instance weighting on the source tasks. (2) the second step uses the reweighted instance to build several SVM classifier models—one model for each hyper-parameter setting. To deal with the second challenge, we present two heuristic methods to select the best set of hyperparameters.

#### 3.3.1. Step-1: instance reweighting

The similarity between the source and target data can be expressed using the similarity in their distributions **P***_S_*(*x*, *y*) and **P**_*t*_(*x*, *y*). Here **P***_S_* represents the joint distribution of all source tasks. Since we do not have access to the labels *y* on the target, we make a simplifying assumption that there is only a covariate shift between the source and target tasks—i.e., the conditional distribution *P*(*y*|*x*) is the same for both tasks. Mathematically, $\frac{P_{S}(x,y)}{P_{t}(x,y)}=\frac{P_{S}(x)}{P_{t}(x)}=r(x)$. Many methods have been proposed for estimating the ratio *r*. Sugiyama et al. (2008) proposed an algorithm Kullback-Leibler Importance Estimation Procedure (KLIEP) to estimate *r* directly without estimating the densities of the two distributions.

We use the nonparametric Kernel Mean Matching (KMM) (Huang et al., 2007), which was originally developed to handle the problem of covariate shift between the training and test data distributions. KMM reweighs the training data instances such that the means of the training and test data distributions are close in a reproducing kernel Hilbert space (RKHS). This approach does not require distribution estimation. Let *x^S^_i_* ~ *P_S_* and *n_S_* be the number of source instances from all source tasks. Let *x^t^_i_* ~ *P_t_* and *n_t_* be the number of target instances. Let β_*i*_ represent the “importance” of the source instances. KMM uses a function based on the *maximum mean discrepancy* statistic (MMD). In the form written below, it minimizes the difference between the empirical means of the joint source and target distributions.

(1) $$\begin{array}{l} \underset{\beta }{\min}\;{\left\|\frac{1}{n_{S}}\overset{n_{S}}{\underset{i=1}{\sum }}\beta_{i}\Phi (x_{i}^{S})-\frac{1}{n_{t}}\overset{n_{t}}{\underset{j=1}{\sum }}\Phi (x_{j}^{t})\right\|}^{2}\\ \;\Leftrightarrow \underset{\beta }{\min}\;\frac{1}{n_{S}^{2}}\beta^{T}K\beta -\frac{2}{n_{S}^{2}}\kappa^{T}\beta \;+C\\ \text{ subject to }\beta_{i}\in [0,B]\text{ and }\sum_{i}\beta_{i}\leq n_{S}\\ \text{ where }K_{i,j}=k(x_{i}^{S},x_{j}^{S})\text{ and }\kappa_{i}=\frac{n_{S}}{n_{t}}\sum_{j=1}^{n_{t}}k(x_{i}^{S},x_{j}^{t}) \end{array}$$

*K* is the kernel matrix over all the source examples. The function (1) is a quadratic program and can be efficiently solved using sequential minimal optimization (SMO), projected gradient based methods. We use the KMM implementation from the Shogun (Sonnenburg et al., 2010) package.

***3.3.1.1. Selecting an appropriate set of source and target instances***. Using all instances in the optimization problem in equation (1) is infeasible for two reasons. The optimization involves the computation of the gram matrix *K* of *O*(*n*^2^) where *n* is the number of instances. Typically the total number of protein-protein pairs between a host-pathogen are of the order of 100 million. Secondly, the total number of labeled source instances is quite small (≈ 1500). This set is likely to get underweighted (i.e., β_*i*_ ≈ 0) if there are too many unlabeled source instances. To represent the source's empirical mean, in addition to the labeled instances we randomly sample four times as many unlabeled instances. For the target, we randomly sampled *n_S_* instances.

#### 3.3.2. Step-2: model learning

Once we have the optimal set of source instances, we can train a Kernel-SVM model using these. Along with the first step, we thus call this two step process KMM-SVM. We pick a kernel-based learning algorithm since we plan to extend our work to deal with different feature spaces across the tasks. In such a scenario, the only mechanism to operate on the target data is via similarities, i.e., the kernel. The dual formulation for the weighted version of SVM solves the following problem, where the weights β_*i*_ were obtained in Step-1.

$$\begin{array}{l} \underset{{}_{\alpha }}{\min}\sum_{i=1}^{n_{S}}\alpha_{i}-\frac{1}{2}\sum_{i,j}\alpha_{i}\alpha_{j}y_{i}y_{j}K(x_{i}^{S},x_{j}^{S})\text{ subject to }\sum_{i}\alpha_{i}y_{i}=0\\ \text{ and }\beta_{i}C\geq \alpha_{i}\geq 0\; \end{array}$$

#### 3.3.3. Model selection

Parameter tuning and selecting the best model in the absence of labeled data is a very hard problem. The model built on the source data cannot be tuned using cross validation on the source data because doing so will optimize it for the source distribution. Hence we developed two heuristic approaches to select the best hyperparameters. The first one uses the expected class-skew on the target task while the second uses reweighted cross-validation.

**Class-skew based parameter selection**: We first built several models by doing a grid-search on the classifier hyper-parameters. There are 3 parameters to tune for the Kernel-SVM: the kernel width γ, the cost parameter *C*, the weight parameter for the positive class *w*_+_. The total number of parameter combinations in our grid-search were 50. We thus had 50 models trained on the reweighted source data obtained after KMM in Step-1 (Section 3.3.1). We applied each model on the target data and computed the predicted class-skew *r_pred_* using the predicted class labels. The expected class skew based on our understanding of the PPI experimental literature is roughly 1:100 (= *r_true_*). We ranked all 50 models on the statistic |*r_pred_* − *r_true_*|. The top *k* models were selected based on this criteria and a weighted voting ensemble was built using them. This ensemble was used to get the final class label on the target data. We used *k* = 5.

**Aggregating the models and assigning interaction scores:** In our experiments, we used *k* = 5 to pick the best models w.r.t the ranking statistic described above. Note that each model gives us a classifier score for every protein-pair in the test data, which can be considered to be the probability of interaction. For *k* = 5, we have five scores for each test protein-pair. These scores were aggregated using two criteria:

- The majority vote over the five models where each model votes “yes” if the output probability score is greater than or equal to 0.5.
- The averaged of all five probability scores.

#### 3.3.4. Spectrum RBF kernel

We used a variant of the spectrum kernel, based on the features used by Dyer et al. (2007) for HIV-human PPI prediction. The kernel uses the *n*-mers of a given input sequence and is defined as: $k_{sp}^{n}(x,x^{\prime })=\exp\{-\frac{\|\phi_{sp}^{n}(x)-\phi_{sp}^{n}(x^{\prime }){\|}^{2}}{\sigma^{2}}\}$, where *x*, *x*′ are two sequences over an alphabet Σ. Instead of using the 20 amino acids as the alphabet Σ, we used a classification of the amino-acids. There are seven classes based on the electrostatic and hydrophobic properties of proteins, i.e., |Σ| = 7. Here ϕ*^n^_sp_* transforms a sequence *s* into a |Σ|^*n*^-dimensional feature-space. One dimension of ϕ*^n^_sp_* corresponds to the normalized frequency of one of the 7*^n^* possible strings in *s*. We use *n* = 2, 3, 4, 5.

## 4. Negative examples and feature-set

Classification techniques need a negative class (set of non-interactions) in order to identify the special characteristics of the positives (i.e., interactions). Since there is no published experimental evidence about “non-interacting” host-pathogen proteins for any plant with *Salmonella*, we construct the negative class using random pairs of proteins sampled from the set of all possible host-pathogen protein pairs. The number of random pairs chosen as the negative class is decided by what we expect the interaction ratio to be. It is a parameter that can be changed as our knowledge of the size and nature of the host-pathogen interactome improves.

The interaction ratio/ negative examples are used in different ways as described below. The homology-based transfer method does not directly use any negative examples/ interaction ratios. In the case of T-SVM, while training the transductive model, we use negative examples from the source task. In the case of KMM-SVM, the data used to build the model comes from the source tasks, where negative examples from each source task are used. Next, during the model selection phase we pick the best models based on the interaction ratio of the model over the predictions on the target task (See Section 3.3.3 for details). No explicit negative examples are used in this part; the interaction ratio is simply used to pick the best model.

We initially chose a positive:negative class ratio of 1:100 meaning that we expect 1 in every 100 random bacteria-human protein pairs to interact with each other. This has been a common practice in host-pathogen PPI prediction in the past (Dyer et al., 2007; Tastan et al., 2009). Recently published work (Mukhtar et al., 2011) involving a yeast-2-hybrid study on plant-bacterial PPIs suggests a higher interaction ratio of around 1:1000. Our choice of 1:100 as the class-skew is an overestimate when considering interactions with all *Salmonella* genes, but if we restrict the binding partners to only the so-called *Salmonella* effector proteins, the ratio we use is reasonable. (There are ≈85 known *Salmonella* effector genes). Also note that, while the exact examples that we choose as negative data may not be true negatives, we expect the false negative rate to be low enough (≈ 1%) to justify our choice of this heuristic.

The class skew is an important parameter in any machine learning method. The choice of this parameter determines the properties of the resultant model. A very balanced class skew of 1:1 will result in a model that is over-predictive i.e., has a very high false positive rate when applied on the target task. On the other hand, a very skewed setting of 1:1000 could give a lower false positive rate but is likely to have a poor recall as compared to models with lower class skews. This parameter thus offers a trade-off between the precision and recall of the resultant model. Our choice of a class ratio of 1:100 will result in a higher recall as compared to models trained on higher class skews. It will however, have some false positives. From a statistical perspective, a model trained with a high class skew such as 1:1000 will capture the distribution of the negatives since they hugely outnumber the positives. Since the negative class examples are not true negatives, the goodness of a model which depends mostly on noisy negatives is debatable. Computationally, the time required for training a model increases as we increase the number of examples. In the case of a high class skew such as 1:1000, there will be thousand times as many examples as the number of positives. This makes training a model very slow, especially for the Kernel-SVM algorithm and Transductive SVM models that are used by our methods. Nonetheless, we also calculated the predictions for a higher skew of 1:500. The results are described in Section 5.

The features used in each approach are shown in Table 1. A detailed description of each feature and the biological significance of it follows. We derive protein sequence based features similar to the ones derived by Dyer et al. (2011) for HIV-human PPI prediction.

- **Protein sequence *n*-mer or *n*-gram features**: Since the sequence of a protein determines its function to a great extent, it may be possible to predict PPIs using the amino acid sequence of a protein pair. Shen et al. (2007) introduced the “conjoint triad model” for predicting PPIs using only amino acid sequences. Shen et al. (2007) partitioned the twenty amino acids into seven classes based on their electrostatic and hydrophobic properties. For each protein, they counted the number of times each distinct three-mer (set of three consecutive amino acids) occurred in the sequence. To account for protein size, they normalized these counts by linearly transforming them to lie between 0 and 1 (see Shen et al. (2007) for details). They represented the protein with a 343-element feature vector, where the value of each feature is the normalized count for each of the 343 (7^3^) possible amino acid three-mers. We use two-, three-, four-, and five-mers. For each host-pathogen protein pair, we concatenated the feature vectors of the individual proteins. Therefore, each host-pathogen protein pair had a feature vector of length at most 98, 646, 4802, and 33614, in the cases of two-, three-, four-, and five-mers, respectively.
- **Gene expression features**: These features depend only on the human protein (gene) involved in a human-*Salmonella* protein pair. We selected 3 transcriptomic datasets from GEO (Barrett et al., 2011), which give the differential gene expression of human genes infected by *Salmonella*. The 3 datasets (GDS77, GDS78, GDS80) give us a total of 7 features representing differential gene expression of human genes in 7 different control conditions. The intuition behind this feature is that genes that are significantly differentially regulated are more likely to be involved in the infection process, and thereby in interactions with bacterial proteins. Note: these were used in only the human-*Salmonella* task.
- **GO similarity features**: These features model the similarity between the functional properties of two proteins. These were used in only the human-*Salmonella* task. Gene Ontology (Ashburner et al., 2000) provides GO-term annotations for three important protein properties: molecular function (F), cellular component (C) and biological process (P). We derive 6 types of features using these properties. For each of “F,” “C,” and “P,” two types of GO similarity features were defined: (a) pair-level similarity and (b) similarity with human protein's binding partners. The similarity between two individual GO terms was computed using the G-Sesame algorithm (Du et al., 2009). This feature is a matrix of all the GO term combinations found in a given protein pair: < *p_s_*, *p_h_* >, the rows of the matrix represent GO terms from protein *p_s_* and the columns represent GO terms from *p_h_*. Analogously, the second feature type-(b) computes the similarity between the GO term sets of the *Salmonella* protein and the human protein's binding partners in the human interactome. We used HPRD to get the human interactome.

**Code**: The executable files from the packages used to build our methods, and the scripts that we used to run these can be downloaded here: http://www.cs.cmu.edu/~mkshirsa/data/frontiers2014/code.zip.

## 5. Results and discussion

A quantitative evaluation on the target task i.e., plant-*Salmonella* is currently not feasible as there is no known PPI data. Hence for the purpose of evaluation, we used some of the PPI datasets as “sources” for building a model and one as the “target.” We evaluate the machine-learning based methods in two settings of transfer: *pathogen-level transfer*, where the host is fixed to be human and the pathogen is one of various bacterial species. The second setting *host-level transfer*, is more relevant and refers to the case where the pathogen is fixed to be *Salmonella* and we modify the host species. Since there are few known PPIs involving *Salmonella*, we are only able to experiment with mouse as an alternate host. There are 14 known mouse-*Salmonella* PPIs. Interestingly they involve mouse proteins whose human homologs also interact with the same *Salmonella* proteins—i.e., these 14 PPIs have interologs in the human-*Salmonella* dataset.

Our evaluation criteria does not use accuracy (which measures performance on both the positives and negatives). Our PPI datasets are highly imbalanced with a large number of negative samples, and a trivial classifier that calls all protein pairs as “negative” will achieve a very good performance. So we instead use precision (P), recall (R) and F-score(F1) computed on the interacting pairs (positive class).

$$\begin{array}{l} \text{ Precision}(\text{P})\;=\frac{\text{true positives}}{\text{predicted positives}};\\ \text{Recall}(\text{R})\;=\frac{\text{true positives}}{\text{total true positives in data}};\\ \text{ F1 score }(\text{F1})=\frac{2PR}{P+R}. \end{array}$$

The source tasks (i.e., training data) and target task (i.e., test datasets) are shown in the Table 2. Parameters for all methods are tuned using a class-skew based model selection similar to the one described in Section 3.3.3 for the KMM-SVM method. We compare the following machine-learning based methods:

1. Inductive Kernel-SVM (Baseline): This model assumes that the source and target distributions are identical. All source data is pooled together and used to build a single model. For the kernel we used the RBF-spectrum kernel.
2. Transductive SVM (T-SVM): This is the method described in Section 3.2.
3. KMM-SVM: This method is discussed in Section 3.3.

**Table 2:** **Performance of the machine learning based methods on various transfer settings**.

**Source task(s) (training data)**	**Target task (test data)**	**Method**	**P^†^**	**R^†^**	**F1^†^**
**HOST-LEVEL TRANSFER**					
*Salmonella*-human	*Salmonella*-mouse	Baseline	42.8	**93.7**	58.8
		T-SVM	45.4	**93.7**	61.2
		KMM-SVM	**51.7**	**93.7**	**66.7**
*Salmonella*-mouse	*Salmonella*-human	Baseline	95.4	33.8	50
		T-SVM	67.5	43.5	**52.9**
		KMM-SVM	**100**	**35.5**	52
**PATHOGEN-LEVEL TRANSFER**					
*Francisella*-human, *E.coli*-human	*Salmonella*-human	Baseline	17.8	12.9	14.9
		T-SVM	15	14.5	14.7
		KMM-SVM	**25.7**	**16.1**	**19.9**
*Francisella*-human, *Salmonella*-human	*E.coli*-human	Baseline	12.9	12.5	12.7
		T-SVM	10.4	15.6	12.5
		KMM-SVM	**15.9**	**21.9**	**18.4**

*We compare them with a simple baseline: inductive kernel-SVM. We report precision (P), recall (R) and f-score (F1). The data that was used to build each of the models is shown in the first column. The second column shows the target task—the data on which we evaluate the model. The numbers in bold font indicate the highest performance in that column (i.e., for that metric)*.

† *Computed using the default classifier threshold: 0.5*.

*The positive:negative class ratio in all datasets was 1:100*.

*The performance of a random classifier would be F-score = 1*.

The host-level transfer performance is shown in the first two rows of Table 2. The KMM-SVM based method performs much better while transferring from *Salmonella*-human to *Salmonella*-mouse. The recall is very high at 93.7 since the mouse-pathogen PPIs are interologs of the human-pathogen PPIs. The precision is not as high as some additional positives are predicted and we found that they had a high classifier score. These “false positives” are likely to be true interactions. For the reverse setting, T-SVM does slightly better than the KMM-SVM and 2 points higher than the baseline. Note that here, the source data is very small in size with only 14 PPIs. In the pathogen-level transfer, on the *Salmonella*-human target task, the F1 of the KMM-SVM method is the highest at 19.9 and is 5 points better than the other two methods. On the *E.coli-human* task, the performance is 18.4 which is 5.7 points better than the other methods.

A very interesting observation to make from the table is, the performance on the target task: *Salmonella*-human in the two settings. In the host-level transfer, the F1 is 52 whereas in the pathogen-level transfer it is much lower at 19.9. The hosts human and mouse are much more similar than the group of bacterial species namely: *Salmonella*, *E. coli* and *F. tularensis*. The source tasks are indeed very critical in determining the performance on the target.

### 5.1. Analysis

We apply the models trained using the procedures from previous sections on *Arabidopsis*-*Salmonella* protein-pairs to get predictions for potential interactions. The homology based approach does not assign any confidence scores to the predictions while both T-SVM and KMM-SVM allow us to obtain a score for every predicted interaction. All predictions from T-SVM with a positive score (>0) are considered to be interacting. For the KMM-SVM method, we filter the predictions using a threshold of 0.7 on the averaged probability-score. (See Section 3.3.3 for details on the probability score computation for the KMM-SVM method). We chose this threshold of 0.7 since all positives in our training data are assigned a score ≥0.7 by the classifier model. The full lists of predicted interactions from all three approaches are available at the following link: http://www.cs.cmu.edu/~mkshirsa/data/frontiers2014/predictions.zip.

The total number of PPI predictions based on the score thresholds described above are: 106,807 for homology-based, 1088 for T-SVM and 163,644 from KMM-SVM. Hundreds of thousands of interacting pairs may not be likely and we therefore expect that many of the predictions are likely to be false positives (FPs). We would like to emphasize that, by ranking the predictions on the classifier scores and picking only the top few we are likely to filter out most of the false positives, since the machine learning models are expected to score FPs lower than the true positives. The threshold of 0.7 for KMM-SVM was chosen just to ensure consistency with the threshold that we observed in the training data (i.e., in the known interactions). If one considers say the top 10% of the predictions from the KMM-SVM method, we have 1636 PPIs over ≈1300 unique *Arabidopsis* proteins and 5 *Salmonella* proteins. Choosing by thresholding the prediction score is one way to select potential interactions for further scrutiny. Another approach is to analyze the predictions based on the biological functions one is interested in. To demonstrate the type of biological functions that are represented in the predictions, we performed GO term enrichment analysis of the *Arabidopsis* proteins involved in the predictions. We can then look at *Arabidopsis* genes with the most enriched GO terms and what their predicted *Salmonella* partners are.

**Figure 5:**
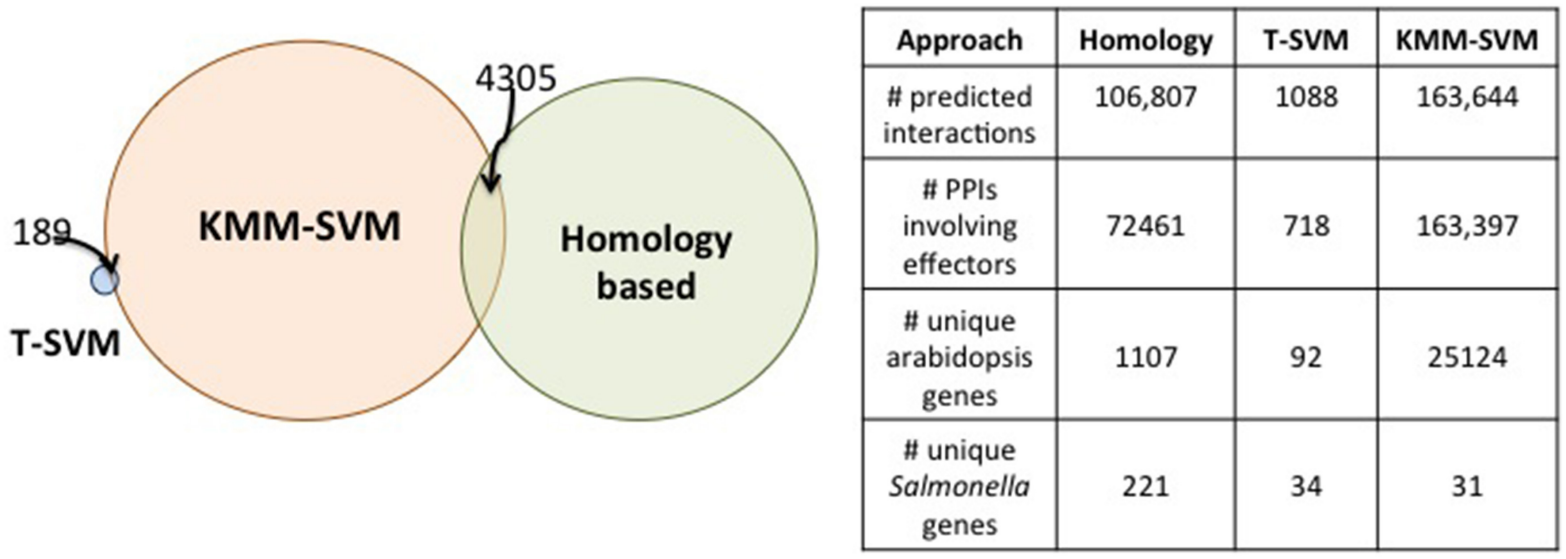
**Overlap amongst the novel PPI predictions from each approach**. All predictions from the homology based approach and the T-SVM are shown. For the KMM-SVM method, we filter the predictions using a threshold of 0.7 on the interaction probability reported by the classifier. We picked this threshold based on the interaction probabilities reported on the known interactions.

A Venn diagram depicting the overlap between the predicted pairs of proteins interacting according to the three approaches is shown in Figure 5. The PPIs reported by each approach are quite different from the others. Only 189 are shared between T-SVM and KMM-SVM and 4305 between the homology approach and KMM-SVM. No overlap was found between the homology approach and the T-SVM approaches. These relatively small overlaps are due to the different input sources (tasks) used by each approach. Further, the machine-learning based approaches KMM-SVM and T-SVM use a discriminative model which employs negative examples whereas the heuristics based approach does not use any such negative data and hence has a small overlap with the other two. The two machine-learning based approaches differ due to the use of different kernels. The KMM-SVM approach is the only approach that shows overlap in predictions to both, the heuristics and the T-SVM approaches, and the results are therefore discussed in detail in the accompanying paper (Schleker et al., 2015).

Because the ratio of 1 positive to 100 negative pairs likely overestimates the number of interactions, we next changed this ratio to 1:500 and generated a new model. As expected, a much smaller number of pairs are predicted namely, 6035. This is a more manageable list and the predictions of the new model are provided at http://www.cs.cmu.edu/~mkshirsa/data/frontiers2014/predictions_class_skew_500.txt.

### 5.2. Qualitative analysis of predicted interactions

As with any predictions, experimental validation is ultimately needed to verify them. The choice depends on the interest of the experimentalist. Here we have chosen for discussion a few predictions that are interesting to us, but we encourage the reader to look at the list of predictions for others of potential biological interest.

We calculated Gene Ontology (GO) enrichment in the *Arabidopsis* proteins predicted to be targeted by the *Salmonella* proteins. We are interested in analyzing the characteristics of the plant proteins predicted to be the most popular targets for pathogenesis. We defined the “popular targets” using the following criteria: (a) the *Arabidopsis* protein is predicted to be targeted by at least 3 *Salmonella* effectors with a probability greater than 0.9 and (b) the GO term annotations of the *Arabidopsis* protein are significantly enriched [with a *p*-value of <0.001 as obtained by GO enrichment analysis using FuncAssociate (Berriz et al., 2003)]. There are a total of 5247 *Arabidopsis* proteins satisfying these criteria. In Table 3, we show 20 *Arabidopsis* genes selected randomly from this set of highly targeted *Arabidopsis* proteins. In Table 4, we show the list of all enriched GO terms.

**Table 3:** **GO terms that were enriched in the most targetted *Arabidopsis* proteins in our predictions**.

**Arabidopsis (TAIR id)**	**Protein name/gene**	**Enriched Gene Ontology annotations**	**Enriched GO terms (corresp. to column 3)**
AT1G01030	B3 domain containing transcription factor	Sequence-specific DNA binding transcription factor activity ; regulation of transcription, DNA-templated	GO:0003700
			GO:0006355
AT1G06160	Ethylene-responsive transcription factor ERF094	DNA binding ; sequence-specific DNA binding transcription factor activity ; regulation of transcription from RNA-polymerase II promoter ; response to jasmonic acid stimulus	GO:0003677
			GO:0003700
			GO:0006355
			GO:0009753
AT1G01060	Myb-related putative transcription factor	Response to cadmium ion ; response to salt stress ; response to auxin stimulus ; response to cold	GO:0046686
			GO:0009651
			GO:0009733
			GO:0009409
AT1G13180	Actin-related protein 3	Actin binding	GO:0003779
AT2G40220	Ethylene-responsive transcription factor ABI4. Protein glucose insensitive 6	DNA binding ; response to water deprivation ; positive regulation of transcription, DNA-dependent ; sequence-specific DNA binding	GO:0003677
			GO:0009414
			GO:0045893
			GO:0043565
AT2G46400	Putative WRKY transcription factor 46	Response to chitin	GO:0010200
AT1G01080	Ribonucleoprotein, putative	nucleic acid binding ; RNA binding	GO:0003676
			GO:0003723
AT3G12110	Actin-11	Chloroplast stroma	GO:0009570
AT3G56400	Probable WRKY transcription factor 70	Response to salicylic acid stimulus ; sequence-specific DNA binding transcription factor activity ; protein amino acid binding	GO:0009751
			GO:0003700
			GO:0005515
AT1G01090	Pyruvate dehydrogenase E1 component subunit alpha-3, chloroplastic	Chloroplast stroma	GO:0009570
AT4G09570	Ca-dependent protein kinase 4	protein amino acid binding	GO:0005515
AT1G01150	Homeodomain-like protein with RING-type zinc finger domain	Zinc ion binding ; regulation of transcription, DNA-templated	GO:0008270
			GO:0006355
AT4G18170	Probable WRKY transcription factor 28	Regulation of transcription, DNA-templated ; sequence- specific DNA binding transcription factor activity	GO:0006355
			GO:0003700
AT1G01160	GRF1-interacting factor 2	Protein amino acid binding	GO:0005515
AT1G01200	Ras-related protein RABA3	GTP binding; small GTPase mediated signal transduction ; protein transport	GO:0005525
			GO:0007264
			GO:0015031
AT5G47220	Ethylene-responsive transcription factor 2	Positive regulation of transcription, DNA-dependent ; ethylene mediated signaling pathway	GO:0045893
			GO:0009873
AT1G01250	Ethylene-responsive TF ERF023	Sequence-specific DNA binding transcription factor activity ; nuclear envelope	GO:0003700
			GO:0005634
AT1G01350	Zinc finger CCCH domain-containing protein 1	Nucleic acid binding ; zinc ion binding	GO:0003676
			GO:0008270
AT1G01370	Histone H3-like centromeric protein HTR12	DNA binding ; protein amino acid binding	GO:0003677
			GO:0005515

*To get this list, we performed a GO term enrichment analysis using the FuncAssociate tool (Berriz et al., 2003). We then procure the set of Arabidopsis genes which correspond to the enriched GO terms; i.e., GO terms with a p-value of <0.001. We further filter this set to include only those Arabidopsis genes predicted to interact with at least 3 Salmonella effector proteins. In this table, we show around 20 such Arabidopsis genes for the lack of space. The remaining are available via the download link*.

**Table 4:** **List of all enriched GO terms obtained by applying enrichment analysis tool FuncAssociate (Berriz et al., 2003) on the set of highly targeted *Arabidopsis* proteins (i.e., *Arabidopsis* proteins predicted to interact with at least 3 *Salmonella* effectors)**.

**GO term**	**Description**
GO:0003676	Nucleic acid binding
GO:0003677	DNA binding
GO:0003700	Sequence-specific DNA binding TF activity
GO:0003723	RNA binding
GO:0003735	Structural constituent of ribosome
GO:0003755	peptidyl-prolyl cis-trans isomerase activity
GO:0003779	Actin binding
GO:0003899	DNA-directed RNA polymerase activity
GO:0004298	Threonine-type endopeptidase activity
GO:0004693	Cyclin-dependent protein serine/threonine kinase activity
GO:0004842	Ubiquitin-protein transferase activity
GO:0004871	Signal transducer activity
GO:0005484	SNAP receptor activity
GO:0005507	Copper ion binding
GO:0005509	Calcium ion binding
GO:0005515	Protein binding
GO:0005525	GTP binding
GO:0005576	Extracellular region
GO:0005622	Intracellular region
GO:0005634	Nuclear envelope
GO:0005839	Proteasome core complex
GO:0005840	Ribosome
GO:0006351	Transcription, DNA-templated
GO:0006355	Regulation of transcription, DNA-templated
GO:0006412	Translation
GO:0006413	Translational initiation
GO:0006457	Protein folding
GO:0006511	Ubiquitin-dependent protein catabolic process
GO:0007264	Small GTPase mediated signal transduction
GO:0007267	Cell-cell signaling
GO:0008233	Peptidase activity
GO:0008270	Zinc ion binding
GO:0008794	Arsenate reductase (glutaredoxin) activity
GO:0009408	Response to heat
GO:0009409	Response to cold
GO:0009414	Response to water deprivation
GO:0009570	Chloroplast stroma
GO:0009579	Thylakoid
GO:0009651	Response to salt stress
GO:0009733	Response to auxin
GO:0009737	Response to abscisic acid
GO:0009739	Response to gibberellin
GO:0009751	Response to salicylic acid
GO:0009753	Response to jasmonic acid
GO:0009828	Plant-type cell wall loosening
GO:0009873	Ethylene mediated signaling pathway
GO:0010200	Response to chitin
GO:0015031	Protein transport
GO:0015035	Protein disulfide oxidoreductase activity
GO:0016491	Oxidoreductase activity
GO:0016607	Nuclear speck
GO:0016762	Xyloglucan:xyloglucosyl transferase activity
GO:0022626	Cytosolic ribosome
GO:0022627	Cytosolic small ribosomal subunit
GO:0042254	Ribosome biogenesis
GO:0042742	Defense response to bacterium
GO:0043565	Sequence-specific DNA binding
GO:0045454	Cell redox homeostasis
GO:0045892	Negative regulation of transcription, DNA-templated
GO:0045893	Positive regulation of transcription, DNA-templated
GO:0046686	Response to cadmium ion
GO:0046872	Metal ion binding
GO:0051726	Regulation of cell cycle

*The shown terms had a p-value less than 0.001*.

For each gene we show the description and the enriched GO annotations. Among the presented *Arabidopsis* proteins, nearly one third are transcription factors. These function e.g., in hormone-mediated signaling pathways. It has been reported that jasmonic acid and ethylene signaling pathways are involved in plant defense response against *Salmonella* (Schikora et al., 2008). Other examples that highlight the role of transcription factors in plant-pathogen interaction are e.g., that a *Xanthomonas* effector protein targets an ethylene responsive transcription factor (ERF) in tomato to inhibit ethylene induced transcription (Kim et al., 2013) and systemic immunity in barley induced by *Xanthomonas* and *Pseudomonas* bacteria may involve WRKY and ERF-like transcription factors (Dey et al., 2014). Further, actin-11 and actin-related proteins involved in actin polymerization and depolymerization are obtained. It is well known that *Salmonella* translocates effectors into the mammalian host cell in order to interact with actin and e.g., modify the cell cytoskeleton to allow bacterial entry (for review see Schleker et al., 2012). Our analysis revealed growth regulating factor 1 (GRF1)-interacting factor 2, a transcriptional co-activator which is part of a regulatory complex with GRF1 and microRNA (miRNA) 396. MiRNAs are involved in plant disease resistance to bacteria and miRNA396 has been shown to be upregulated in plants upon flg22 treatment (Li et al., 2010). Liu et al. (2014) reported that putative GRF1 targets in *Arabidopsis* are heavily involved in biosynthetic and metabolic pathways, e.g., phenylpropanoid, amino acids and lignin biosynthesis as well as plant hormone signal transduction indicating the role of GRF1 in plant defense mechanisms. Other examples of predicted interactions and more details of their possible relevance in *Salmonella*-plant interplay are discussed in the accompanying paper (Schleker et al., 2015).

### 5.3. Limitations and future work

In this paper, we addressed the challenge of predicting the *Salmonella*-*Arabidopsis* interactome in the absence of any experimentally known interactions. Previous work in this area was based purely on homology between human and *Arabidopsis* proteins and was therefore limited to proteins that do display sequence similarity. Due to the large divergence between the two organisms, this approach neglects a large fraction of potential *Arabidopsis* targets. We therefore presented here three different sophisticated computational and machine learning methods to predict hereto unknown *Salmonella*-plant interactions from a relatively small list of known *Salmonella*-human interactions. This is a very challenging task because it is not possible to quantitatively validate the predictions. Nonetheless, the predictions provide a gold-mine for discovery because they provide experimentally testable hypotheses on the communication mechanisms between plant and *Salmonella* without restriction to known effectors in the pathogen or sequences of similarity to those observed in better studied eukaryotic organisms. With these advantages comes a set of limitations to be aware of.

Since machine learning methods need some known interactions to evaluate the models on, and to pick the best set of predictions, their application in the current paper has limitations. For example, we can obtain different predictions from our methods by varying the parameters, especially the class skew (we studied the ratios 1:100 and 1:500 in this paper). Because there are currently no known *Salmonella*-plant interactions, we are not able to quantify which of these sets of predictions is more reliable. Augmenting the predictions with some other biological information from the target task can help in picking the most plausible PPIs. This is a direction for future research. Further,

1. The interactome predicted by each method is not the true interactome, but is a set of predictions. There will be false positive and false negative interactions. Thus, each individual prediction has to be considered a hypothesis not a fact.
2. In line with point 1 above, the size of the predicted interactomes does not necessarily relate to the true interactome. We dont know how many interactions to expect. Our different predictions vary greatly in size, with one method predicting only one thousand interactions, while others predict more than 100,000 interactions. While it is more likely that smaller numbers of interactions are more likely, it does not mean that this method is inherently better than the other methods.
3. The size of the predicted interactions list also depends on a critical parameter, the positive to negative class ratio. This parameter is important but it is tuneable, so the methods validity is not dependent on its choice. However, it is important to appreciate that the predictions will differ greatly when this parameter is changed. Thus, biological insight in choosing predictions to validate still needs to be applied, regardless of the prior choice of ratio in generating the model.

These general limitations in the context of the specific results of the models presented here translate to the following issues, pointed out by a reviewer of this paper: The data presented for the KMM-SVM model indicate that 163,644 PPIs are predicted (Figure 5). This is of the same order of magnitude as the number of false positives that would be predicted, given the reported false positive rate of the method that indicate ≈180,000 false positive PPIs would be expected. This raises the possibility that the bulk of the predictions may be false positives. The data presented for the KMM-SVM model also indicates that 25,124 distinct *Arabidopsis* genes participate in PPIs with 31 distinct *Salmonella* genes (Figure 5). This implies that 91% of the *Arabidopsis* protein-coding gene complement (TAIR10: 27,416 genes— http://www.arabidopsis.org/portals/genAnnotation/gene_structural_annotation/annotation_data.jsp) enters into productive interaction with only 31 *Salmonella* proteins. It also implies that, on average, each interacting *Salmonella* protein is capable of productive interaction with over 5000 *Arabidopsis* proteins. It is unlikely that this is the case, again suggesting that a large number of false positives have to be expected.

### Conflict of interest statement

The authors declare that the research was conducted in the absence of any commercial or financial relationships that could be construed as a potential conflict of interest.
